# The neuroinflammatory role of microglia in Alzheimer's disease and their associated therapeutic targets

**DOI:** 10.1111/cns.14856

**Published:** 2024-07-19

**Authors:** Melika AmeliMojarad, Mandana AmeliMojarad

**Affiliations:** ^1^ Department of Bioprocess Engineering, Institute of Industrial and Environmental Biotechnology National Institute of Genetic Engineering and Biotechnology Tehran Iran

**Keywords:** Alzheimer's disease, microglia, neuroinflammation, NSAIDs

## Abstract

**Introduction:**

Alzheimer's disease (AD), the main cause of dementia, is characterized by synaptic loss and neurodegeneration. Amyloid‐β (Aβ) accumulation, hyperphosphorylation of tau protein, and neurofibrillary tangles (NFTs) in the brain are considered to be the initiating factors of AD. However, this hypothesis falls short of explaining many aspects of AD pathogenesis. Recently, there has been mounting evidence that neuroinflammation plays a key role in the pathophysiology of AD and causes neurodegeneration by over‐activating microglia and releasing inflammatory mediators.

**Methods:**

PubMed, Web of Science, EMBASE, and MEDLINE were used for searching and summarizing all the recent publications related to inflammation and its association with Alzheimer's disease.

**Results:**

Our review shows how inflammatory dysregulation influences AD pathology as well as the roles of microglia in neuroinflammation, the possible microglia‐associated therapeutic targets, top neuroinflammatory biomarkers, and anti‐inflammatory drugs that combat inflammation.

**Conclusion:**

In conclusion, microglial inflammatory reactions are important factors in AD pathogenesis and need to be discussed in more detail for promising therapeutic strategies.

## Graphical abstract

The main hallmarks of AD are leading to neuron loss and neuron degeneration in AD.

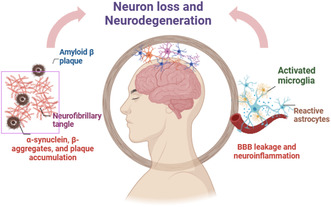

## 1 BACKGROUND

Alzheimer's disease (AD); a toxic neurodegenerative disorder is the most common cause of dementia and is becoming more common in the elderly.^1^ The World Health Organization (WHO) projects that 132 million people worldwide will have dementia by the year 2050.^2^, ^3^ Progressive neuropathological processes in AD include neurodegeneration and loss of neuronal synapses, which are primarily brought on by an increase in amyloid‐β (Aβ) plaques accumulation and hyperphosphorylated tau protein.^4^, ^5^ Different risk factors have been detected for AD development such as age, cardiovascular changes, metabolic disorders, increased metal ions accumulation, and brain injury.^4^, ^6^ Even though amyloid plaques and neurofibrillary tangles play a deadly role in the brain, the abnormal inflammatory mediators produced by microglia, a type of important glial cells linked to the innate immune response,^7^, ^8^, ^9^ is one of the new hallmarks of neuroinflammation in AD.^10^

Microglia cells interact with tau and Aβ, Toll‐like receptor (TLR), colony‐stimulating factor‐1 receptor (CSF1R), and receptor expressed on myeloid cells 2 (TREM2) are all activated by glial activation through the signaling pathways of apolipoprotein E (ApoE).^11^, ^12^, ^13^

Although the initial activation of microglial cells activation by Aβ deposition is a helpful reaction for phagocytosing the burden however, extended activation of microglial cells diminishes their capacity for Aβ phagocytosis and induces the release of proinflammatory cytokines, reactive oxygen species, and nitric oxide, which causes chronic inflammation and worsens AD conditions.^7^, ^14^, ^15^

Therefore, microglial cells with their dual role may offer new therapeutic targets that could prove to be just as significant as the amyloid and tau proteins that constitute the plaques and tangles themselves.^16^ Currently, nonsteroidal anti‐inflammatory drugs (NSAIDs) and cholinesterase inhibitors are approved drugs to delay AD but none of them could cure the disease.^17^, ^18^ Therefore, identification of core pathologies mechanism responsible for AD and potential therapeutic targets is essential.^19^, ^20^ In this review, we focused on an in‐depth evaluation of microglia and their AD‐associated inflammatory factors as a new interest target of AD pathogenesis research. In addition, we highlighted the top anti‐inflammatory therapeutic approach and inflammatory biomarkers for AD treatment.

## 2 MICROGLIA AND M1 TO M2 SWITCH

Microglia are innate immune cells derived from myeloid tissues found in the central nervous system (CNS),^21^ playing critical functions in homeostasis, neuron protection, development, differentiation, survival, synaptic plasticity, and neuronal metabolism.^22^

Through pathological circumstances and the BBB damage, circulating monocytes can penetrate the brain parenchyma and colonize the microglial niche.^23^ Recent studies indicate the role of circulating monocytes in AD pathology.^23^, ^24^ Through the adoption of various regulatory networks, microglial activity is thought to synchronize with the CNS's development, maturation, and senescence throughout life. Microglial activity also plays significant roles in immune surveillance, neuronal apoptosis, and maintaining synaptic plasticity.^15^, ^25^ Microglia‐inflammatory responses, inducing neuroinflammation are one of the most notable characteristics of many neurodegenerative diseases, such as Parkinson's disease, AD, and amyotrophic lateral sclerosis.^26^

There is growing evidence that the central nervous system exhibits heterogeneous microglial activation, which can be divided into two opposing types: M1 phenotype and M2 phenotype.^26^, ^27^ The cytotoxic or neuroprotective effects of microglia are dependent on the phenotypes that are activated.^26^, ^27^

M2 microglia induction results in elevated secretion of anti‐inflammatory factors while, M1 microglia induction is responsible for the damage of neurons and oligodendrocytes through the secretion of pro‐inflammatory cytokines including TNF, IL‐1, and nuclear factor κB (NF‐κB).^26^, ^28^ M1 microglia typically predominate at the site of injury during the final stages of the disease, when M2 microglia's ability to resolve immune responses and heal themselves is diminished.^26^ In addition to supporting tissue repair and extracellular matrix reconstruction, M2 microglia also facilitate the phagocytosis of misfolded proteins cell debris, and neurotrophic factors that support neuron survival.^26^ Consequently, boosting microglial cells' M2 polarization or the balance between microglia M1/M2 polarization could be a useful treatment approach for AD.^26^, ^29^

## 3 THE BLOOD–BRAIN BARRIER (BBB) AND INFLAMMATION

By maintaining a stable and well‐regulated microenvironment in the central nervous system (CNS), the BBB is one of the primary tight brain barriers that shields the brain from harmful substances.^23^ It also shields peripheral pro‐inflammatory cytokines, toxins, and infectious agents.^30^ Peripheral inflammatory mediators, immune and inflammatory cells that cross the BBB, such as mast cells and T cells, as well as pro‐inflammatory mediators released by AD microglia cause disruption of the BBB.^31^, ^32^, ^33^

## 4 MICROGLIA AND NEUROINFLAMMATION

The activation process of Microglia is followed by a series of morphological and biological functions leading to the release of pro‐inflammatory mediators and phagocytic activity.^20^, ^34^, ^35^ In response to the Aβ peptides, microglia release cytotoxic factors that facilitate the phagocytosis of these peptides and aid in their clearance.^36^ Thus, while phagocytosis of amyloid‐β peptides may help the disease progress, the release of proinflammatory mediators appears to exacerbate the condition.^36^

**FIGURE 1:**
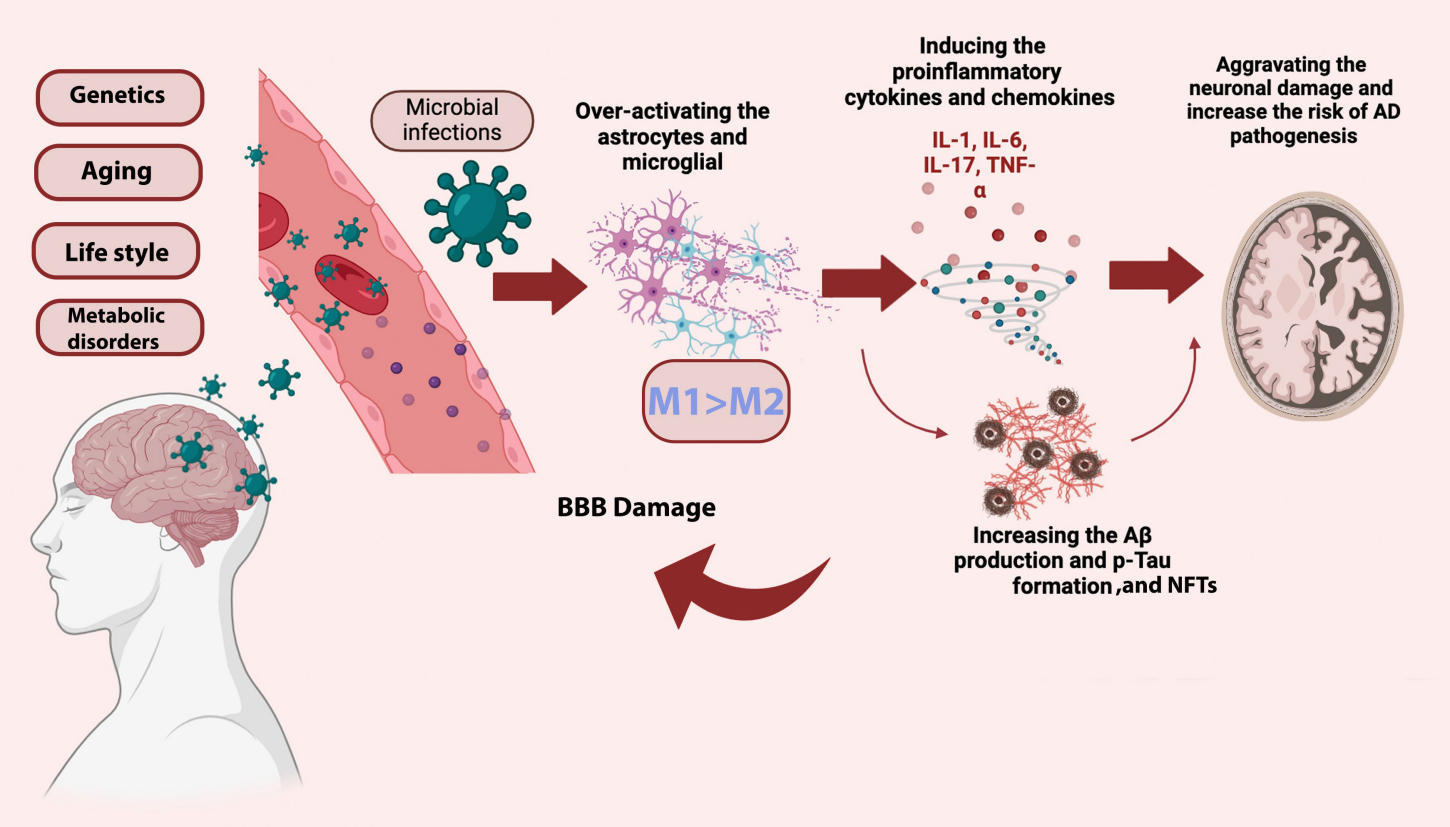
Activated microglia cell responses leading to AD pathology. Besides the two common risk factors leading to AD, including the extra‐accumulation of amyloid‐β plaques and tau phosphorylation, microglial cells are among the main pathogenic hallmarks of AD via inducing the M1 phenotype modification and increased release of the inflammatory cytokines causing BBB leakage. In addition, BBB damage can increase the entrance and activity of other immune cells in the brain over‐activating other rested microglia cells in the brain and triggering them to produce more inflammatory mediators including cytokines the inflammatory cascades, which increase the extracellular plaques accumulation and leading to neurodegeneration and loss of neuronal synapses.

M1 and M2 polarization switches play the most significant role in the proper activation of microglia and release of pro‐inflammatory mediators such as reactive oxygen species (ROS), nitric oxide (NO), IL‐1, IL‐6, TNF‐α, IFN‐γ to increase phagocytosis.^35^, ^37^, ^38^ However, microglia activation appears to have a beneficial effect on Aβ clearance during the chronic phase of neuroinflammation and early AD development by phagocytosis.^17^, ^39^ As shown in Figure 1, the persistent brain stimulation of microglia in response to Aβ plaques accumulation, tau protein phosphorylation, and inflammatory responses may advance AD pathogenesis and cause neuroinflammation.^9^ As AD worsens, prolonged activation of microglia reduces their capacity to phagocytose, generates pro‐inflammatory mediators, and aggravates tau and Aβ pathology.^40^ Mutations in microglia‐related genes have a substantial impact on the ability of microglia, causing them to become permanently activated, reducing their capacity for phagocytosis, and ultimately resulting in neuroinflammation and neurodegeneration.^16^ Therefore, understanding the molecular mechanism of microglia is highly important to detect their dual roles in either Aβ plaques accumulation or degradation.^6^, ^41^

Recently, certain molecular regulators of microglial proliferation have been directly demonstrated to exist including triggering receptors expressed on myeloid cells 2 (TREM2) and apolipoprotein E (APOE) which are both among AD risk factors for late‐onset AD (LOAD).^42^ In the central nervous system, APOE4 plays multiple roles, such as maintaining lipid homeostasis, healing damaged neurons, eliminating toxins like Aβ, and immune response modulators.^43^ Among all APOE isoforms, APOE4 has been shown to exacerbate tau‐mediated neurodegeneration, while the absence of APOE4 is protective in Patients with AD.^44^ Patients who carry at least one APOE4 allele show faster disease progression, and increased brain atrophy compared to non‐APOE4 carriers.^45^, ^46^ As previously mentioned, it inhibits the gene that produces SIRT1, a molecule that has been associated with longer lifespans and has anti‐Alzheimer's properties instead It's linked to nuclear factor kappa B (NF‐κB) activation, which encourages inflammation.^45^, ^47^ The Aβ binding to APOE4 and other apolipoproteins was tested in different in vitro.^16^, ^45^ Even though the binding was consistently verified, none of those investigations suggested that variations in APOE‐Aβ binding were linked to an increased risk of AD.^10^, ^45^ According to Yuan et al., TREM2 deficiency increased the amount of diffuse amyloid plaques that covered a greater surface area due to longer and more branched amyloid fibrils.^48^ Through TREM2 binding, APOE evaluates the phagocytosis and APOE‐Aβ uptake, while the TREM2 R47H variant has less affinity to bind with APOE.^12^, ^49^ Due to its dysregulation of neuroinflammation and elevation of AD risk, the missense mutation R47H of TREM2 is linked to AD risk.^50^ A dose‐dependent reduction in TREM2 inhibits the accumulation of myeloid cells surrounding Aβ plaques. In addition, plaques number and size are decreased in TREM2 deficiency.^40^, ^51^

Microglia in plaques‐loaded brain areas of AD transgenic mice expressed more TREM2, suggesting a significant role for TREM2 against AD.^48^ Growing data indicates that TREM2 deficiency supports microglial phagocytosis and maintains microglial responses to Aβ deposition by inhibiting the transition of microglia from a homeostatic to a disease‐oriented state.^40^, ^52^ TREM2 in blood and CSF can act as biomarkers for the diagnosis of early AD since, the TREM2 levels in CSF increase in the early stages of AD, while it decreases in late stages.^40^

Besides the two last popular AD hallmarks, recent data suggest that the fractalkine ligand and its microglial receptor (CX3CL1/CX3CR1) can influence pathologies related to tau by controlling microglial migration and attracting monocytes to the brain.^53^ It was determined through repeated examination of the brains of AD‐afflicted humans and animals that activated; phagocytic microglia encircle amyloid plaques.^54^ Microglia most likely proliferate more quickly and assemble around fibrillar amyloid plaques because of dysregulated fractalkine/CX3CR1 signaling brought on by CX3CR1 receptor deletion, indicating that CX3CR1 has been found to maintain microglia in an inactive, non‐neurotoxic condition.^54^, ^55^, ^56^ Mice deficient in CX3CR1 showed an altered inflammatory milieu, decreased neuronal loss, and an increase in the amount of Aβ phagocytosis mediated by microglia however aggravated tau phosphorylation was also detected.^57^

Similarly, colony‐stimulating factor 1 receptor (CSF1R) inhibition has attenuated the neurodegeneration process caused by tau proteins.^13^ Mutation of IFNγ receptors increases Aβ synthesis and microglial activation.^58^, ^59^

The CSF‐1‐CSF‐1R pathway, which is mainly active in reactive microgliosis conditions, has also been connected to microglia survival in the context of TREM2 expression.^21^, ^60^ This pathway affects Aβ clearance. A similar mechanism may also be involved in microglial survival, as it has been shown that TREM2 promotes macrophage survival via the CSF‐1R pathway.^13^ The role of CSF‐1R signaling in microglia survival is detected by a study indicating that TREM2‐deficient microglia exhibit reduced survival at low CSF‐1 concentrations.^61^

The genetic deletion of the inflammatory NLR family pyrin domain containing 3 (NLRP3) facilitates the synthesis of IL‐1β and improves Aβ clearance by microglia as well as cognitive function in AD mice.^62^, ^63^ NLRP3 activation increases AD pathogenesis by damaging the microglia mitochondrial aggregation and impairs the structural and functional integrity of mitochondria by increasing the release of proinflammatory cytokines.^64^ Top genes related to microglia activity are listed in Table 1.

**TABLE 1:** Topgenes related to microglial activity and their functions in AD.

Gene	Function	Expression	Reference
Microglia genes in Aβ pathogenesis			
SR‐A	Regulation of microglia phagocytosis	Increased in AD	41
CD36	Regulation of microglia phagocytosis	Increased in AD	65
RAGE	Regulation of microglia phagocytosis	Increased in AD	66
*APOE*	Regulation of microglia phagocytosis	Increased in AD	67
*CR1*	Modulate microglia phagocytosis of Aβ	Increased in AD	68
*CD33*	Modulate microglia phagocytosis of Aβ	Increased in AD	69
*TREM2*	Modulate Aβ phagocytosis	Decreased in AD	50
*ABCA7*	Modulate microglia phagocytosis of Aβ	Increased in AD	70
Microglia genes in neuroinflammation			
NLRP3	Modulate microglia‐mediated inflammatory response	Increased in AD	71
BACE1	Increasing inflammatory responses	Increase in AD	72
SOCS	Regulate the balancing of inflammatory response	Decreased in AD	50
SHIP1	Modulate microglia‐mediated inflammatory response	Decreased in AD	73
CX3CR1	Regulate *tau phosphorylation*	Decreased in AD	74
Microglia genes in tau pathology			
CSF1R	Modifying tau‐mediated neurodegeneration	Increased in AD	21
APOE	Modifying tau‐mediated neurodegeneration	Increased in AD	45, 67
TREM2	Regulating Aβ plaques and tau aggregates	Decreased in AD	75

## 5 INFLAMMATION AND AD

Numerous clinical investigations point to the involvement of inflammation in cognitive decline, particularly in the pathogenesis of AD.^76^ Apart from amyloid‐beta (Aβ) protein accumulation and hyperphosphorylated tau protein, inflammation is currently thought to be the third primary hallmark of AD.^55^, ^77^ The two main groups of molecules that cause inflammation are transcription factors and cytokines.^78^ While the inflammatory response can help by speeding up the clearance of Aβ, it can also cause an increase in tau and Aβ synthesis, which can lead to neurodegeneration and synapse loss.^78^ The timely elimination of invasive pathogens and the cessation of an overreaction within the central nervous system are guaranteed by the equilibrium between the initiation and termination of the immune response and are essential for preventing a variety of illnesses, such as AD.^79^ The expression of genes involved in regulatory neuronal function may be permanently altered by the improper activation of inflammatory cytokines.^31^ For instance, cytokines can negatively impact the synaptic plasticity required for synapse formation and activity‐dependent synaptic pruning by interacting with various immune molecule groups, such as major histocompatibility complex class I (MHC I).^80^ These modifications in synaptogenesis are thought to be essential to dementia's causes.^81^ Furthermore, cytokines have the power to significantly activate the hypothalamic–pituitary–adrenal (HPA) axis and raise hormone release.^82^

The BBB can be influenced and penetrated by pro‐inflammatory cytokines that lead to chronic inflammation, such as TNF‐α, IL‐6, and IL‐1β. This can cause the BBB to release proinflammatory mediators and increase cell permeability, which allows leukocytes to enter the brain.^83^, ^84^ In addition, cytokines that reduce inflammation are generated. These consist of IL‐4, IL‐10, IL‐11, and IL‐1 receptor antagonist. These cytokines might be a component of an intricate system that guards against exaggerated neuroinflammation.^85^, ^86^ When the NF‐κB pathway is activated in microglia, tau seeding and spreading can increase, and most AD patients have significantly higher levels of NF‐κB.^87^ Homeostatic and cognitive abnormalities were restored along with the silencing of microglial NF‐κB. Thus, a potential therapeutic strategy to reduce AD pathogenesis is to inhibit the NF‐κB pathway.^88^ Ultimately, an additional factor that may either directly or indirectly exacerbate inflammation and neuroinflammatory mediators is the excessive generation of neutrophil extracellular traps (NETs), which trigger tissue damage and macrophage activation.^82^, ^89^ Consequently, chronic inflammation may result from the long‐term activation of microglia and astrocytes. Infections with bacteria and viruses, aging, and certain environmental factors can also result in chronic inflammation.^90^ A significant alteration in the activation of inflammatory pathways occurs in chronic inflammation, resulting in altered immune responses and an overabundance of inflammatory cytokines that cause neuroinflammation.^91^

## 6 INFLAMMATORY BIOMARKERS AND AD

The two primary fluid‐based biomarkers of cerebrospinal fluid (CSF) used in clinical practice at the moment are phosphorylated tau proteins and Aβ42.^92^ However, there are still limitations in their specific detection based on their low concentration in blood.^14^, ^93^ As previously indicated, inflammation plays a significant role in the development of AD, and of all the neuroinflammatory biomarkers that can be used as therapeutic targets for drug development, cytokines, and transcription factors have garnered a lot of attention due to their precise roles in the various stages of AD and potential medical applications.^29^, ^79^ Results regarding the levels of numerous cytokines in patients with different diseases were obtained from different research groups. There is evidence that several cytokine levels, which are also useful as inflammatory markers, are rising in AD, including IL‐1β, TNF‐α, and NF‐κB.^79^, ^92^

The finding that fast‐progressing AD is associated with an IFN‐γ polymorphism suggests that cytokines, particularly LOAD, may actively contribute to the disease's acceleration.^94^, ^95^ Additionally, dysregulation of the cytokines can impact the phenotype of microglia and hasten the progression of AD.^79^ Tumor necrosis factor (TNF)‐α, IL‐6, MCP‐1, interleukin (IL)‐1β, and IL‐6 are among the inflammatory cytokines and chemokines secreted from activated microglia.^16^, ^89^

However, the most recent meta‐analysis found significant heterogeneity in some comparisons but no discernible differences between AD patients and healthy controls in terms of cytokines like IL‐1β, IL‐6, IL‐8, IL‐10, or TNF‐α.^9^, ^96^

Thus, more investigation is required to ascertain whether cytokines and AD are associated synergistically. Furthermore, due to damage to the BBB, various proteins can cross it; as a result, the blood of AD patients may reveal targets linked to the disease's progression. More significantly, the BBB's vast surface area presents a therapeutic intervention opportunity.^97^, ^98^

Consequently, other techniques for identifying AD include the identification of well‐established inflammatory biomarkers and early diagnosis and patient monitoring techniques. However, given the unpredictable outcomes of using a single cytokine level, it makes more sense to combine the use of several proteins to demonstrate that an imbalance in cytokine levels is the sole cause of AD. Cytokines may not be sufficient in this regard. However, since the first AD prediction model consisting of 18 plasma biomarkers with multiple cytokines was proposed, only a small number of sets of biomarkers have shown consistent performance and good reproducibility. Hypersensitive techniques like single‐molecular mass analysis (SIMOA) and immunoprecipitation‐mass spectrometry (IP‐MS) can identify even the smallest variations in the Aβ plasma level in AD patients.^99^, ^100^

A more sensible strategy is to use multiple proteins in combination.^94^, ^101^ However, since the first AD prediction model consisting of 18 plasma biomarkers with multiple cytokines was proposed, only a small number of biomarker sets have shown consistent performance and good reproducibility.^101^

Moreover, screening 120 inflammatory molecules in CSF and serum of AD, MCI, and healthy controls using protein‐array analysis revealed that soluble IL‐6 receptor (sIL‐6R), tissue inhibitor of metalloproteinases‐1 (TIMP‐1), and soluble TNF‐α receptor I (sTNFR‐I) in CSF provided the best prediction to AD among other molecules.^102^, ^103^ Future studies in AD should investigate pathogens other than Aβ and the role that cytokines play in interactions with other players. The establishment of brain banks is the only way to find new genes and proteins, and genome‐wide association studies and online database analysis will continuously update polymorphism data associated with AD.^102^, ^104^ To the best of our knowledge, we highlighted all the inflammatory biomarkers for the treatment strategy of AD is provided in Table 2.

**TABLE 2:** The highlight of top neuroinflammatory biomarkers for AD.

Marker	Function in inflammation	References
IL‐1α and IL‐1β	Increased in CSF of AD patients	105
ICAM‐1	Increased in CSF of AD patients	106
VCAM‐1	Increased in CSF of AD patients	107, 108
TNF‐α	Increased in serum and CSF of AD patients	109
IL‐6	Increased in serum and CSF of AD patients	31
IL‐12	Increased in serum and CSF of AD patients	110
NF‐κB	Transcription factor that activates genes related to inflammation	95, 111
CCL2	Increased in serum and CSF of AD patients	112
IL‐8	Increased in serum and CSF of AD patients	113
IL‐33	Increased in plasma of AD patients	114, 115
Progranulin	A neuroinflammatory modulator for early prediction of AD patients	116
YKL‐40	Increasing the neuroinflammation in astrocytes	110, 117

## 7 THERAPEUTIC STRATEGIES FOR AD

Recent discoveries linking inflammation and neurodegeneration are opening up new treatment options.^99^

Reducing the generation and accumulation of toxic Aβ plaques as well as the inflammatory responses is currently a key treatment approach for AD.^8^, ^63^ NSAIDs are frequently used medications for AD because they can reduce the amount of Aβ plaques, microglial activation, and proinflammatory cytokine levels by inhibiting the production of cyclooxygenase (COX), and other important substances in AD pathogenesis.^118^ Eight classes of NSAIDs can be distinguished by their chemical makeup and degree of inhibition selectivity.^118^ Of note, there are COX‐1 and COX‐2 are the two primary COX isoenzymes, and as was previously mentioned, they have different expression patterns and functions. It is generally accepted that NSAID classes 1–7 are non‐selective and inhibit both COX‐1 and COX‐2, whereas class 8 NSAIDs inhibit COX‐2. The COX‐2 inhibitors celecoxib and rofecoxib, which lessen AD neuroinflammation, are currently the most promising medications for reducing inflammation,^18^, ^119^ even though there is currently no known treatment for the disease.^120^

COX‐2 inhibitors eliminate the cyclooxygenase (COX‐1 and COX‐2) enzyme, needed for the transformation of arachidonic acid into prostacyclin or prostaglandins, which has a degenerative effect and is capable of raising Aβ levels.^18^, ^19^

Interestingly, it has been observed that high levels of COX‐2 are expressed by degenerative brain cells; this suggests that blocking COX may mitigate AD. Direct or indirect Aβ‐induced microglial activation can increase COX‐2, which is present during inflammation.^18^

Compared to control brains, AD brains exhibit higher levels of COX‐1 and COX‐2.^120^ NSAIDs may be beneficial in the fight against AD, according to research employing animal models of the disease. For example, in transgenic mice overexpressing APP, oral administration of ibuprofen, a nonspecific COX inhibitor, at the onset of amyloid plaque formation decreased glial activation and plaque density.^120^

Numerous epidemiological investigations, randomized controlled trials (RCTs), and meta‐analyses have examined the possible therapeutic benefit of NSAIDs in AD, with conflicting findings.^121^ While the majority of clinical trials were negative, some epidemiological studies and meta‐analyses suggested that NSAIDs had a beneficial effect on the progression of AD.^18^, ^81^ According to Nilsson et al., high‐dose aspirin can potentially have an impact even at low doses by reducing the development of AD (OR 0.41, 95% CI 0.26–67).^122^ However, the majority of research suggests that for an effect to be proven, longer exposure to NSAIDs is necessary.^122^, ^123^ Furthermore, it is crucial to stress that NSAIDs' protective benefits become apparent with prolonged use, particularly in the early stages of AD intervention, as it is likely that NSAIDs will lose their effectiveness once well‐established AD lesions have developed.^123^ Overall, NSAIDs were thought to be associated with a decreased risk of AD based on recent observational studies.^123^

In another Double‐blind, placebo‐controlled study with 160 participants, indomethacin slows down the progression of cognitive decline in AD patients.^124^

In a separate study, rats given indomethacin had less microglial activation, a longer‐lasting improvement in the hippocampus, and no issues with working memory. Moreover, mice that received an intracerebroventricular injection of Aβ showed increased COX‐2 levels.^18^, ^125^

In addition, pretreatment with the specific COX‐2 inhibitor NS398 reduced COX‐2 levels and cognitive impairment.^126^

Additional research has shown that treatment with ibuprofen and naproxen in AD‐model transgenic mice There is hope for the use of NSAIDs in the treatment of AD based on additional research on human cell cultures.^125^, ^127^, ^128^

For instance, the overexpress APP695NL, in human neuroglioma cells identified different NSAIDs that can selectively reduce Aβ42 such as sulindac, ibuprofen, and diclofenac.^20^, ^129^ Another potential neuroprotective mechanism of NSAIDs is through activating PPARγ, a transcriptional factor that inhibits the expression of proinflammatory genes by blocking the activity of other transcription factors like NFκB, AP‐1, and STAT1.^130^ Furthermore, PPARγ can inhibit proinflammatory genes in the vasculature and myeloid lineage cells such as macrophages and microglia.^130^, ^131^, ^132^, ^133^

As a result, clinical AD research has used pioglitazone, a PPARγ agonist, to suppress the expression of genes that promote inflammation to regulate transcription.^134^ However, the use of NSAIDs is only advantageous during the initial phases of AD, as they become dangerous and ineffective as the Aβ deposition process progresses because they reduce microglial inflammation, which mediates the clearance of A despite its deleterious effects.^135^

Another tactic to combat AD and AD‐related inflammatory responses is to target the NLRP3 inflammasome of microglia. Small molecule NLRP3 inhibitors like JC‐124 and MCC950 have been found to strongly promote pro‐inflammatory cytokines, chemokines, and ROS in AD; however, these along with more thorough analyses of the results may yield delightfully unexpected outcomes.^16^, ^136^, ^137^, ^138^

A tetracycline with anti‐inflammatory properties that can pass through the BBB is minocycline.^139^ According to an in vivo study, minocycline inhibits the NLRP3 inflammasome, which lowers Aβ accumulation and attenuates microglial activation.^139^, ^140^ As a dihydropyridine calcium channel antagonist, nicodipine (P2X7R antagonists) has also been demonstrated to provide neuroprotective effects by lowering activated NF‐κB levels and preventing the release of mature IL‐1β in Aβ‐stimulated microglia (whose possible target is P2X7R), which is permissive in NLRP3 inflammasome activation and cytokine synthesis.^141^, ^142^, ^143^ To the best of our knowledge, we highlighted all the agents for therapeutic strategies in AD in Table 3.

**TABLE 3:** The top current agents for therapeutic strategies in AD.

Drug	Targets	Function	References
Minocycline	NLRP3 inflammasome blockage	Attenuating microglial activation and reduces Aβ accumulation	140
MCC950	NLRP3 inflammasome blockage	Attenuating microglial activation and reduces Aβ accumulation	144
JC‐124	NLRP3 inflammasome blockage	Attenuating microglial activation and reduces Aβ accumulation	138, 145
Ibuprofen	NLRP3 inflammasome blockage	Attenuating microglial activation and reduces Aβ accumulation	125
Edaravone	NLRP3 inflammasome blockage	Attenuating microglial activation and reduces Aβ accumulation	146
P2X7Rinhibitor	NLRP3 inflammasome blockage	Attenuating microglial activation and reduces Aβ accumulation	142
P22	CD33 inhibitor	Boosting Aβ phagocytosis	147
Lintuzumab	CD33 inhibitor	Boosting Aβ phagocytosis	148
4D9 antibody	TREM2 Modulator	Boosting microglial phagocytosis	40
AL002c	TREM2 Modulator	Neuroprotective effects via reducing Aβ	149
AL002a	TREM2 Modulators	Neuroprotective effects via reducing Aβ	75
Ethyl pyruvate	Toll‐like receptor 4 (TLR4) inhibitor	Cognitive improvement	150
Thymoquinone	Toll‐like receptor 4 (TLR4) inhibitor	Cognitive improvement	151
TAK‐242	Toll‐like receptor 4 (TLR4) inhibitor	Cognitive improvement	151
TLR4	Toll‐like receptor 4 (TLR4) inhibitor	M2 Microglial Polarization Induction	152
JN‐J527	CSF1R inhibitor	Functional improvement	61
GW2580	CSF1R inhibitor	Recovery of short‐term memory	153
PLX3397	CSF1R inhibitor	Suppress tau propagation	154
PLX5622	CSF1R inhibitor	Prevent plaques formation	21

## 8 CONCLUSION

Chronic inflammation is currently considered the third core pathology in the initiation and progression of AD, besides the well‐known studied functions of Aβ and tau. microglia cells in the central nervous system play essential roles in this inflammatory process. Activated Microglia is considered as the main source of cytokines release and triggering the inflammatory cascades in the CNS which consequently, causes loss of synapses and neurodegeneration, and increases the levels of p‐tau and Aβ. Since there are still no effective therapies in terms of disease attenuation or prevention, further research is needed to unrevealing the potential inflammatory targets and to evaluate their effects on microglia over‐activation and AD mechanisms. Finding trustworthy biomarkers is crucial for monitoring AD. By offering anti‐inflammatory treatment strategies for AD, the utilization of a multiplex model through the combination of various inflammatory biomarkers and anti‐inflammatory drugs we may improve the precision of AD diagnosis and encourage the identification of biomarker levels in the blood.

## AUTHOR CONTRIBUTIONS

Melika AmeliMojarad and Mandana AmeliMojarad wrote the main manuscript text and prepared figures. All authors reviewed the manuscript.

## CONFLICT OF INTEREST STATEMENT

The authors declare no conflicts of interest.

## Data Availability

The data that support the findings of this study are available from the corresponding author upon reasonable request.
